# Standing Posture in Motor and Cognitive Dual-Tasks during Smartphone Use: Linear and Nonlinear Analysis of Postural Control

**DOI:** 10.3390/ejihpe12080073

**Published:** 2022-08-09

**Authors:** Marina Saraiva, Orlando J. Fernandes, João Paulo Vilas-Boas, Maria António Castro

**Affiliations:** 1 RoboCorp Laboratory, i2A, Polytechnic Institute of Coimbra, 3046-854 Coimbra, Portugal; 2 Faculty of Sports and CIAFEL, University of Porto, 4200-450 Porto, Portugal; 3 Sport and Health Department, School of Health and Human Development, University of Évora, 7000-671 Évora, Portugal; 4 Comprehensive Health Research Center (CHRC), University of Évora, 7000-671 Évora, Portugal; 5 LABIOMEP—Porto Biomechanics Laboratory, Faculty of Sports and CIFI2D, University of Porto, 4200-450 Porto, Portugal; 6 Centre for Mechanical Engineering, Materials and Processes (CEMMPRE), University of Coimbra, 3030-788 Coimbra, Portugal; 7 School of Health Sciences—Physiotherapy, Polytechnic Institute of Leiria, 2411-901 Leiria, Portugal

**Keywords:** dual-task, center of pressure, approximate entropy, linear analysis CoP, smartphone use, standing posture

## Abstract

Analysis of the center of pressure (CoP) during cognitive or motor dual-tasking is widely used to characterize postural control. Most studies use traditional measures of CoP to quantify postural control, but given its complexity, nonlinear analysis of CoP is of growing interest in the area. This study aims to analyze CoP behavior in healthy young adults during standing posture performance while simultaneously performing motor or cognitive tasks on a smartphone, using linear and nonlinear analysis of CoP. Thirty-six healthy participants (23.08 ± 3.92 years) were found eligible for this study. They performed a single task (ST), cognitive dual-task (cog-DT), and motor dual-task (mot-DT). The total excursion of CoP, displacement of CoP in the anterior-posterior and medial-lateral directions, mean total velocity of CoP, and mean anterior-posterior and medial-lateral velocities of CoP were measured with a force plate. Approximate entropy (ApEn) of the anterior-posterior (ApEn-AP) and medial-lateral (ApEn-ML) displacement of CoP were also calculated. The results showed that dual-task costs for the total excursion, displacement in the anterior-posterior direction, mean total velocity, and mean anterior-posterior velocity of CoP were greater during the cog-DT than the mot-DT (*p* < 0.05). In the nonlinear analysis of the CoP, there was no difference (*p* > 0.05) between the cog-DT and mot-DT for ApEn values of the anterior-posterior and medial-lateral time series of the CoP. Both linear and nonlinear analyses showed differences between the cog-DT and ST (*p* < 0.05), revealing a decline in postural control during the cog-DT compared with the ST. In conclusion, performing a cog-DT causes sway impairments and lower postural control efficacy compared with motor single and dual-tasks. Furthermore, both linear and nonlinear analyses were able to distinguish between conditions.

## 1. Introduction

Postural control refers to the ability to maintain, reach, or restore a state of balance during any posture or activity [1]. The ability to stand upright on two feet is a prerequisite for initiating other activities, and provides essential information about balance and the postural control system [2].

During human quiet standing, the center of pressure (CoP)—the point of application of the ground reaction force vector—is constantly readjusted to achieve human balance and counteract the body’s sway. For this reason, the motion of the CoP is the measure most often used to assess postural sway during static postural control [3]; although some authors assess balance performance using the center of gravity, measuring the center of pressure by force plate is considered the gold standard for assessing postural balance [4,5].

There are two main approaches to assessing CoP behavior: linear and nonlinear analysis. Linear analysis describes the quality of movement, the magnitude and/or variance of the CoP displacements (e.g., range of CoP sway, velocity of CoP, ellipse sway area), whereas nonlinear measures provide information about the temporal organization of the variation in CoP displacement regarding motor behavior over time [6]. This variability is intrinsic within all biological systems, and an important characteristic of adaptive postural behavior, reflecting variations in time and space [7]. Approximate entropy (ApEn) is one of several measures of nonlinear analysis of the CoP. ApEn is a system complexity and regularity measure that quantifies the randomness in a time series in various situations [8]. It is a useful measure of postural sway complexity in an experimental time series and has been used to describe changes in postural control [9,10]. ApEn values range between 0 (more regular sway) and 2 (irregular and unpredictable sway) [8,11]. Smaller approximate entropy values are associated with a lower complexity of structure and more regular and predictable CoP signals, whereas higher values indicate larger irregularities in the CoP, being more random and less predictable. The lower complexity of physical movements shows a higher rigidity and lower flexibility of postural control, whereas higher complexity is translated as enhanced self-organization and effective strategy in postural control [12].

Maintaining an upright posture while performing one or more concurrent tasks is common in daily activities. For example, using different smartphone functions (e.g., listening to music, sending or reading messages, talking, web surfing, and playing games) while standing, walking, or working [13]. Using the dual-task, it is possible to assess the effects of concurrent motor or cognitive tasks on motor performance and the attentional demands of a motor task [14]. Simultaneously executing two tasks demands a higher level of attention, balancing ability, and executive function compared with a single-task performance [15]. Generally, when performing simultaneous tasks, there is a decline in performance for one or both tasks, which is referred to as dual-task interference (DTI) [16].

The performance decline in dual-tasks has been demonstrated in several studies, showing a decrease in postural stability under cognitive or motor dual-task conditions in healthy individuals (young and older people) and neurological patients (e.g., Parkinson’s disease, multiple sclerosis, etc.) through sway analysis (traditional CoP analysis) [17]. The smartphone is an electronic device massively used worldwide by all ages, and its use is associated with pedestrian accidents [18] and physical and psychological problems [19,20]. However, studies that have considered smartphone use as a secondary task when assessing postural stability are limited, particularly in studies where the primary task is standing posture [21].

Based on entropy analysis, previous studies that have assessed cognitive dual-task performance during standing have suggested that the regularity of CoP trajectories is positively correlated with the amount of cognitive involvement in postural control [22,23]. This means less cognitive involvement in postural control yields less regular postural sway (higher entropy) when introducing a cognitive task [22,23].

Most studies analyzed the effect of mobile phone use on postural control while walking. They found that mobile phone use negatively compromises gait kinematics (e.g., gait speed, stride length, stance phase, and cadence) and gait stability [24,25]. For this reason, we analyzed the effect of performing cognitive and motor dual-tasks involving smartphone use on static postural control; once that, many of the functions used on the smartphone involve motor and cognitive tasks. 

It is important to characterize and understand postural control stability and motor control mechanisms in healthy young adults when performing different tasks in quiet standing posture to predict falls and postural control impairments. The linear analysis of CoP displacements is the usual assessment of postural control in an upright stance, although data is not interpreted from a physiological point of view [26]. The nonlinear analysis adds this perspective, as it assesses the flexibility and capacity of the postural control system to adapt to an unpredictable and constantly changing environment [27]. Thus, we added the nonlinear analysis to characterize the dynamic organization of CoP displacements during a dual-task in an upright stance because it is a complex task representing the sum of various neuromusculoskeletal systems [28]. Moreover, standing posture is fundamental to adequately performing other tasks and movements [28,29]; therefore, it is pertinent to assess the regularity and stability of the CoP in health systems to predict diseases or impairments in postural control.

To the best of our knowledge, few studies have used approximate entropy in CoP time series analyses during dual-task performance [30,31,32], especially when maintaining a quiet standing posture while using a smartphone [33]. Thus, using linear and nonlinear analysis, we aimed to analyze CoP behavior in standing posture performance while simultaneously performing motor or cognitive tasks on the smartphone in healthy young adults to identify which of the tasks interfered most with postural control performance. We hypothesized that: (1) Young adults would have lower postural control performance when performing a cognitive task on their smartphone while maintaining a standing posture than when performing a secondary motor task (dual-task interference); (2) There would be lower complexity of postural control and greater center of pressure kinematic impairments in cognitive and motor dual-tasks than in a single task.

## 2. Materials and Methods

An a priori power analysis was conducted using G*power software (Franz Faul, Edgar Erdfelder, Axel Buchner, Universität Kiel, Kiel, Germany, version 3.1.9.6) to calculate the necessary sample size [34]. With α = 0.05 and a power of 0.95, a minimum of 24 individuals was needed to achieve a large effect size (d = 0.8).

Thirty-six healthy young adults between 18 and 35 years of age participated in this study (see sample characteristics in Table 1). They were medication-free, had no neurological, vestibular, visual, musculoskeletal, or cardiorespiratory dysfunctions, and no active disease at the time of data collection. They gave written informed consent for participation in this study, which was approved by the Ethics Committee of the Polytechnic Institute of Coimbra (approval number: 27_CEPC2/2019) and conformed to the Declaration of Helsinki.

### 2.1. Postural Control Assessment

Subjects were instructed to quietly stand upright on a force plate to perform all tasks. Ground reaction forces and moments were recorded using a model FP4060-07-1000 Bertec^®^ force plate (Bertec Corporation, Columbus, OH, USA) with a sampling frequency of 100 Hz. These measures were later used to compute the coordinates of the center of pressure in the anterior-posterior (CoP-AP) and medial-lateral (CoP-ML) axes. We smoothed the signals using a second order low pass Butterworth filter with a cut-off frequency of 50 Hz. Postural control has been characterized by measures of the magnitude and variation of displacements, such as the total excursion of the CoP (TOTEX CoP), displacements of the CoP in medial-lateral (CoP-ML) and anterior-posterior (CoP-AP) directions, mean total velocity of CoP (MVELO CoP), and mean anterior-posterior (MVELO CoP-AP) and medial-lateral (MVELO CoP-ML) velocities of CoP during task performance.

The algorithm for calculating ApEn begins with the time series data of length N with an embedding dimension, m (pattern length), and a lag. The time series of length N is divided into short vectors of length [11]. The ApEn algorithm was calculated by applying the following Equation (1):

(1) $${ɸ}_{m}={\left(N-m+1\right)}^{-1}\overset{N-m+1}{\underset{i=1}{\sum }}\log\left(N_{i}\right)$$

After power spectral analysis, the approximate entropy was calculated using the initial data file. We calculated separate ApEn values for the anterior-posterior (ApEn-AP) and medial-lateral (ApEn-ML) components of the CoP coordinate time series. Values of m of 2 or 3 and r ranging from 0.1 to 0.3 have been recommended to analyze the ApEn of physiological signals. The selection of the parameters m = 2 and r = 0.15 were commonly used to calculate the Approximate Entropy of CoP data [35,36,37]. Given a time series of length N, ApEn (m, r, N) is approximately equal to the negative average natural logarithm of the conditional probability that two subseries of length m are similar (within a tolerance given by ± r times the standard deviation of the time series). We used m = 2 and r = 0.1.

### 2.2. Single Motor Task

A single task was used as baseline control. All subjects were instructed to naturally stand upright on a force plate and relax without smartphone use for 60 s (standing posture) [38,39].

### 2.3. Cognitive Dual-Task

The cognitive dual-task consisted of keeping a quiet standing posture while performing a concurrent cognitive task: playing a cognitive game based on arithmetic or memory tasks (cog-DT) on their smartphone for 60 s. The arithmetic task consisted of a sum or subtraction calculation with one or two digits. The participants were instructed to verbalize their responses to neutralize the motor component (typing on the smartphone). The memory task consisted of memorizing three different elements (a number, the color of the number, and an image), and then repeating the memorized elements for a few seconds. The cognitive tasks described involve similar cognitive processes and can be classified in the same category [40]. For each participant, the cognitive task was randomly chosen.

### 2.4. Motor Dual-Task

The participants were instructed to keep a quiet standing posture while performing a concurrent motor task: typing on the smartphone keyboard (mot-DT). They were informed to type randomly on the smartphone keyboard to neutralize the cognitive component (e.g., not thinking in words or constructing sentences or texts).

Each participant repeated each task once, with a 45 s rest period between tasks. No priority was given to cognitive, motor, and standing postural tasks. The participants were instructed to use their smartphone and hold it as they usually did while playing a game (cognitive task) and typing on the smartphone keyboard, maintaining this position and regular smartphone manipulation for an ecological analysis.

### 2.5. Dual-Task Cost (DTC)

The following Equation (2) [41] was used to identify which of the secondary tasks interferes most with postural control performance. The DTC represents the percentage of changes in CoP behavior from the single task (ST, baseline) to cognitive and motor dual-task (DT) conditions:

(2) $$\%\;DTC\;\left(outcome\right)=\frac{DT-ST}{ST}*100\;$$

The DTC was calculated for CoP linear outcomes (DTC_CoP_) and ApEn (DTC_ApEn_) in both dual-tasks, cognitive dual-task costs (cog-DTC), and motor dual-task costs (mot-DTC).

Higher positive DTC values represent a greater percentage of change from ST to DT in CoP linear outcomes, signifying worse postural control during dual-task performance than single-task performance. On the other hand, in ApEn analysis, negative DTC values represent lower complexity and more regular postural sway (lower entropy), which was found when performing dual-tasks compared with the ST.

### 2.6. Statistical Analysis

The statistical analysis was performed using IBM-SPSS 25.0 software. Quantitative descriptive data related to sample characteristics, CoP linear measures and DTC values were reported as mean ± SD (standard deviation); the ApEn data were presented as median values. Homogeneity of variance and normality of the distribution for the parameters were verified using Levene’s and Shapiro–Wilk tests, respectively. Some outcomes did not have a normal distribution; thus, these data were assessed using non-parametric tests. The differences in CoP linear outcomes and ApEn between motor and cognitive DTCs were examined with the related samples Wilcoxon signed-rank test, to determine which of the secondary tasks interfered most with postural control performance.

The stabilometric data analysis among the three conditions (single task, motor, and cognitive dual-tasks) was performed with the Friedman test and post-hoc Bonferroni corrections to analyze CoP behavior in standing posture performance while simultaneously performing motor or cognitive tasks.

The statistical significance level was set at *p* < 0.05.

## 3. Results

During the dual-tasks, most participants held the smartphone with both hands; there were no differences in CoP values between participants who held the smartphone with one versus two hands (*p* > 0.05).

### 3.1. Dual-Task Interference

**Figure 1:**
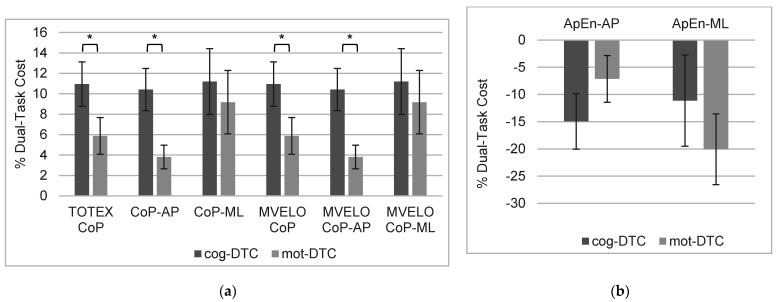
Means and standard errors (error bars) for the cognitive and motor dual-task costs in CoP (**a**) linear and (**b**) nonlinear outcomes. Cog-DTC, cognitive dual-task cost; mot-DTC, motor dual-task cost; TOTEX CoP, total excursion of the center of pressure; CoP-AP, displacement of the center of pressure in anterior-posterior direction; CoP-ML, displacement medial-lateral direction; MVELO CoP, mean total velocity displacement of CoP; MVELO CoP-AP, mean velocity displacement anterior-posterior of CoP; MVELO CoP-ML, mean velocity displacement medial-lateral of CoP; ApEn-Ap, Approximate entropy for anterior-posterior components of the CoP coordinate time series; ApEn-ML, Approximate entropy for medial-lateral components of the CoP coordinate time series. * *p*-value < 0.05: Wilcoxon signed-rank test (using median values): cog-DTC compared with mot-DTC.

Figure 1 shows the results obtained for the cognitive and motor dual-task costs and the differences between both dual-task costs in CoP linear outcomes and ApEn. Cognitive and motor dual-task cost results in CoP linear outcomes showed a decrease in postural control performance when simultaneously performing cognitive or motor tasks while maintaining a quiet standing posture compared with performing a single task. The cognitive dual-task cost for CoP linear outcomes was superior to the motor dual-task cost values. Differences between cognitive and motor dual-task costs were observed in the total excursion of the CoP (*p* = 0.027), displacement of the CoP in the anterior-posterior direction (*p* = 0.002), mean total velocity of CoP (*p* = 0.027), and mean anterior-posterior velocity of CoP (*p* = 0.002). However, there were no differences between cognitive and motor DTC in the displacement of the CoP in the media-lateral direction and mean media-lateral velocity of CoP (*p* > 0.05).

Negative DTC values were found in ApEn for the anterior-posterior and medial-lateral components of the COP coordinate time series, showing a decrease in entropy from the single task to the cognitive and motor dual-tasks. However, there were no differences between cog-DTC_ApEn_ and mot-DTC_ApEn_ (*p* > 0.05).

### 3.2. CoP: Linear and Nonlinear Analysis 

**Figure 2:**
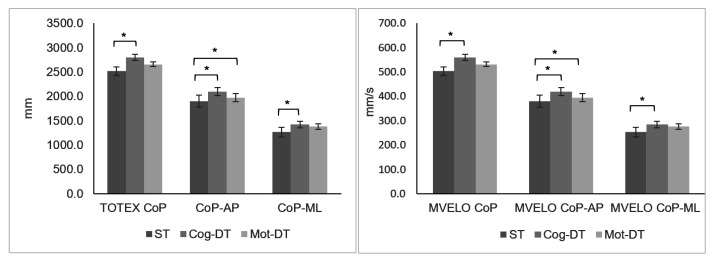
Mean and standard errors (error bars) for each CoP linear outcome during the single task, cognitive, and motor dual-task performance. ST, single task; cog-DT, cognitive dual-task; mot-DT, motor dual-task; TOTEX CoP, total excursion of the center of pressure; CoP-AP, displacement of the center of pressure in anterior-posterior direction; CoP-ML, displacement medial-lateral direction; MVELO CoP, mean total velocity displacement of CoP; MVELO CoP-AP, mean velocity displacement anterior-posterior of CoP; MVELO CoP-ML, mean velocity displacement medial-lateral of CoP. * *p*-value < 0.05: Friedman test with Bonferroni correction for multiple comparisons.

**Figure 3:**
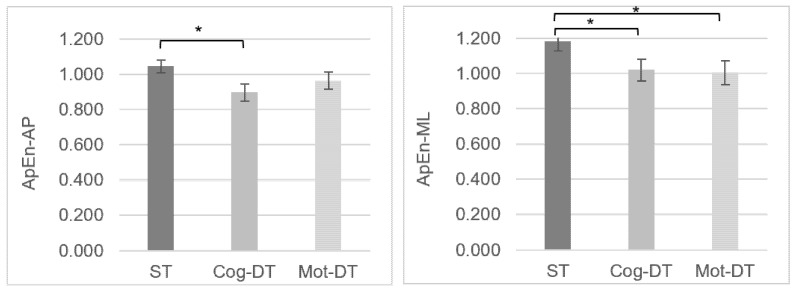
Comparisons of CoP behavior among tasks using nonlinear analysis: approximate entropy (median values and standard errors (error bars)). ApEn-AP, approximate entropy for anterior-posterior components of the CoP coordinate time series; ApEn-ML, approximate entropy for medial-lateral components of the CoP coordinate time series; ST, single task; Cog-DT, cognitive dual-task; Mot-DT, motor dual-task. * *p*-value < 0.05: Friedman test with Bonferroni correction for multiple comparisons.

The CoP behavior in standing posture performance with simultaneous performance of motor or cognitive tasks through linear and nonlinear analysis is presented in Figure 2 and Figure 3, respectively.

Means of the total excursion of the CoP, displacements of the CoP in medial-lateral and anterior-posterior directions, mean total velocity of CoP, and mean anterior-posterior and medial-lateral velocities of CoP increased from single-task to motor and cognitive dual-task conditions (Figure 2). Between the single task, motor, and cognitive dual-tasks, there were differences in each CoP linear outcome (TOTEX CoP: *p* < 0.001; CoP-AP: *p* < 0.001; CoP-ML: *p* = 0.001, MVELO CoP: *p* < 0.001; MVELO CoP-AP *p* < 0.001; MVELO CoP-ML; *p* = 0.001). Post-hoc analysis showed differences in all CoP linear outcomes between the single task (i.e., maintaining a quiet standing position without a smartphone) and cognitive dual-task (i.e., maintaining a quiet standing position while concurrently performing a cognitive task on the smartphone). The differences in CoP-AP were also found between the ST and motor dual-task (i.e., maintaining a quiet standing position while random typing on the smartphone keyboard). For each CoP linear outcome, no differences were found between the cognitive and motor dual-tasks (*p* > 0.05).

CoP nonlinear analysis showed a decrease in the ApEn-AP and ApEn-ML time series values from the single task to both dual-tasks (Figure 3); the difference between the three tasks was significant (ApEn-AP: *p* = 0.009; ApEn-ML: *p* < 0.001). The cognitive and motor dual-task performance caused lower complexity and greater regularity in the center of pressure sway (smaller ApEn values) than the single task.

Post-hoc analysis (Figure 3) showed no difference between cognitive and motor dual-tasks for ApEn-AP and ApEn-ML time series values (*p* > 0.05). However, differences were found between the ST and cog-DT for ApEn-AP (*p* = 0.007) and ApEn-ML (*p* = 0.003) time series values and between ST and mot-DT for ApEn-ML time series values (*p* < 0.001).

## 4. Discussion

In the present study, we used linear and nonlinear analysis of the center of pressure to investigate center of pressure behavior (dual-task interference) between a cognitive dual-task (i.e., maintaining a standing posture while performing a cognitive task on the smartphone) and a motor dual-task (i.e., maintaining a standing posture while randomly typing on a smartphone keyboard). In addition, we also analyzed CoP behavior using linear and nonlinear analysis comparing a quiet standing posture without smartphone use and quiet standing posture while concurrently performing cognitive or motor tasks on a smartphone.

The dual-task costs of the total excursion, displacement in the anterior-posterior direction, mean total velocity displacement, and the mean anterior-posterior velocity of the CoP were higher during the cognitive dual-task than during the motor dual-task. This suggests that the cognitive dual-task was more challenging than the motor dual-task and caused greater perturbations on postural control in healthy young adults. In addition, the cognitive and motor DTC values for the ApEn showed lower complexity and greater regularity in the center of pressure sway (smaller ApEn values) than in the single task, suggesting a decrease in postural stability during both dual-task conditions. However, no significant difference was found between the cog-DTC and mot-DTC for ApEn.

When we examined postural control performance between the single task and cognitive and motor dual-tasks, the linear and nonlinear data showed that postural control performance was inferior under dual-task compared with single-task conditions. The total excursion of the CoP, displacements of the CoP in medial-lateral and anterior-posterior directions, mean total velocity of CoP, and mean anterior-posterior and medial-lateral velocities of CoP increased from single-task to motor and cognitive dual-task conditions. ApEn-AP and ApEn-ML time series values decreased from the single task to both dual-tasks. However, the differences were seen to be more consistent between the cognitive dual-task and single task.

Dual-task performance requires integrity of the cognitive process and challenging attentional capacities, such as sharing attention between tasks [42]. Therefore, participants may have had difficulty maintaining standing sway during the cognitive dual-task, compared with the motor dual-task and single task, because of the inadequate division of attention between two tasks (capacity sharing theory) [43]. Thus, during the cognitive dual-task, brain regions needed to recruit more cognitive resources to perform the task than in the motor dual-task, due to greater cognitive effort and the prefrontal cortex’s role in executive function. Some studies using neuroimaging techniques showed higher frontal lobe activity when subjects performed cognitive tasks compared with motor tasks [44]; others showed an increase in prefrontal cortex activity when performing a cognitive dual-task compared with a single task [45,46]. 

Some tasks (e.g., sitting, standing, or walking) that we judge to be automated require cognitive processing. Thus, postural control is negatively affected during a dual-task, such as maintaining balance while simultaneously performing a second attentionally demanding cognitive or motor task [47,48]. Our results suggest that young adults prioritized the smartphone tasks, performing secondary tasks rather than maintaining higher postural stability (primary task). Makisako et al. [49] found that cognitive tasks had a greater impact than motor tasks on increasing anterior-posterior trunk acceleration during a Romberg stance in older people compared with young adults.

Our findings suggest that the cognitive load and verbalization of responses inherent to the cognitive task can explain the increase in postural sway compared with the secondary motor task. Previous studies showed an increase in postural sway in healthy individuals when performing a spoken mental arithmetic task due to the effect of articulation rather than the cognitive activity [50]. Another study found an increase in sway area, velocity, and length of sway path of the center of pressure during a verbal task, attributing these findings to the increased respiratory muscle activity during vocalization [51]. Indeed, increasing respiratory frequency increases fluctuations in the displacement of CoP in healthy young adults [52].

Earlier studies reported that texting negatively affected postural stability during walking and quiet standing in healthy young adults [33,53]. Nurwulan et al. [33] assessed static and dynamic postural control (normal and tandem stance, and star excursion balance tests, respectively) with and without a smartphone (texting messages), using traditional CoP analysis (total excursion, mean displacement velocity, and sway area of CoP) and nonlinear analysis of CoP (multivariate multiscale entropy). They found higher values for stabilometric parameters of traditional CoP and a smaller value for multivariate multiscale entropy when maintaining a normal stance while texting, compared with only maintaining a normal stance, supporting the theory that a secondary task perturbs postural stability. 

Another study evaluated the influence of speaking on the phone versus texting on postural balance performance in healthy young adults and concluded that both secondary tasks, when simultaneously performed during a quiet standing posture, caused an increase in the center of pressure path length, 90% confidence area, and maximum CoP speed when compared with the control task (quiet standing posture without smartphone use). This study also reported that talking on the phone affected postural stability more than texting a message [39].

Our results from the nonlinear analysis of the CoP were consistent with our linear data. They showed that when cognitive or motor dual-tasks were performed, the approximate entropy decreased compared with the single task, suggesting a lower effectiveness of postural control and greater regularity on the center of pressure during dual-task performance. However, no differences were found between the cog-DTC and mot-DTC for ApEn values. On the other hand, when we compared the postural control performance of the single task with the cognitive and motor dual-tasks, there were significant ApEn-Ap and ApEn-ML differences between the cognitive dual-task and single task. Only the ApEn-ML differences were significant between the motor dual-task and single task. These data support previous findings that showed that approximate entropy could detect changes in postural control in young adults [30]. Donker et al. [22] showed that the regularity of CoP was positively correlated with the attentional demand invested in postural control and, in some situations, increasing internal focus could impair postural control. According to our ApEn results, participants were focused on performing the secondary task (motor or cognitive tasks), leading to a loss of motor system complexity during both dual-tasks.

In this study, the ApEn-AP values were higher for the motor dual-task compared with the cognitive dual-task (no significant difference), which demonstrates that motor dual-task performance may lead to greater automatic postural control and complex and irregular sway than cognitive dual-tasks. This suggests a reduced adaptive capacity of postural control during cognitive dual-task performance [54]. The automatization of postural control during a motor dual-task may be due to how often people communicate via text messaging; thus, spending more time on this task [55].

Other studies that evaluated the spatio-temporal structure of CoP oscillation using other methods of entropy analysis found higher entropy values in cognitive dual-tasks compared with single tasks (standing posture). For example, Kuczyṅski et al. [32] evaluated balance (CoP) using sample entropy during a quiet stance (single task) and a cognitive dual-task in competitive dancers and non-dancers. They found an increase in sample entropy in dual-tasks compared with single-task performance for both groups, showing no interference of the cognitive task on postural control and higher postural stability in the dual-task. Stins et al. [31] analyzed CoP fluctuations in health children and children with higher levels of anxiety while maintaining a quiet stance and simultaneously performing a cognitive task; they found a higher sample entropy in healthy children compared with the anxiety group during the cognitive dual-task, demonstrating greater regularity of the CoP time series on children with higher levels of anxiety. However, between the single (standing) and cognitive dual-task, the sample entropy was slightly lower during cognitive dual-task performance in both groups. 

Our results showed a more regular pattern for CoP variability and reduced postural control stability during dual-task performance compared with the single task; however, the method for entropy analysis (ApEn) differed between these studies (sample entropy and multivariate multiscale entropy) and the demands of the secondary tasks may have contributed to the different entropy results.

It was difficult to compare our results with other studies because there have been few studies assessing CoP behavior on standing posture while using a smartphone (dual-task) using entropy analysis [33]. Some studies analyzed postural control during dual-task performance using entropy analysis in individuals with diseases [31,56], and there have been different methodological approaches to measure entropy in postural control beyond approximate entropy (e.g., Shannon entropy, Renyi entropy, sample entropy, multiscale entropy). This can influence results and entropy data interpretations [26]. 

The ApEn is strongly dependent on record length, which can create a bias toward low ApEn values for shorter time series [12,57]. Our data collection lasted 60 s for each task. Other studies that assessed ApEn collected data for a shorter duration, such as 30 s [58,59]. We suggest that future studies compare different data collection times to provide an adequate record length for entropy analysis. The motor system uses different strategies for postural stability [60]. Thus, we also recommend studies that use the same methodology and analyze postural control using other nonlinear measures to better understand the postural control’s behavior or adaptive capacity during the dual-task performance. 

In previous studies that evaluated the influence of smartphone use on static or dynamic postural control, the baseline postural task was performed without smartphone use [33,61,62,63]. For our baseline task, we also used a single task without smartphone use; however, this task could be considered a limitation of this study because the head positions during the single and dual-tasks were different. The head position was in neck flexion (forward head posture) during smartphone use in the cognitive and motor dual-tasks, and this may have contributed to greater variations in the CoP between the single and the dual-tasks. This may explain the differences in behavior of the CoP between tasks. Furthermore, previous studies have found associations between the stabilometric values of CoP and head position in the frontal plane, reporting an increase in postural instability caused by an increase in the head inclination angle in the frontal plane [64,65]. Thus, analyzing head and neck posture while performing dual-tasks with smartphone usage could be relevant for understanding the effects of head posture on center of pressure behavior. Another limitation could be due to the effect of verbalizing involved in the cognitive dual-task, which could have further influenced CoP behavior. In addition, the respiratory frequency was not controlled, which could have altered CoP displacement.

Future studies are recommended to clarify the influence of verbal tasks on CoP behavior, as the effects of verbalization and the cognitive task are unclear. In other words, it is important to determine which action, talking or the cognitive task, is responsible for the increase in oscillation of the CoP.

The greater regularity of the CoP time series reveals postural control that is more constrained due to mechanical stiffness or neurophysiological impairment [66]. Furthermore, during the dual-task, muscle activity decreased, suggesting there was less attentional processing capacity available to maintain postural control during the dual-task performance, in both older and young adults [67]. For this reason, we suggest integrating the analysis of muscle activity during tasks using electromyography to better understand the mechanisms involved in postural control.

Smartphone use is associated with physical and mental health problems. Our results showed that when young adults performed a cognitive or motor task on a smartphone while maintaining a standing posture, they compromised their postural control performance. Therefore, clinical recommendations should be made to improve postural control under dual-task conditions, such as dual-task training with associated smartphone tasks.

## 5. Conclusions

Maintaining a quiet standing posture while performing a cognitive task on the smartphone appears to be more challenging than maintaining postural stability while performing a motor task.

The present study also suggests that performing cognitive or motor tasks while using a smartphone impairs similar oscillations of CoP during standing posture compared with single-task performance in young adults. However, the cognitive task increased body sway during a standing posture significantly more than during the single task.

Cognitive dual-task performance caused greater impairment of CoP linear outcomes and greater regularity in the center of pressure; consequently, there was less efficacy in static postural control compared with the motor dual-task and single task conditions in healthy young adults. 

Both linear and nonlinear methods were able to highlight the effects of dual-tasks on CoP stability.

## Figures and Tables

**Table 1:** Anthropometric and demographic characteristics and smartphone use data of the sample (mean ± SD).

Variables	Sample (n = 36)
Age (years)	23.08 ± 3.92
Height (m)	1.71 ± 0.10
Body mass (Kg)	73.99 ± 15.97
BMI (Kg/m^2^)	25.15 ± 4.37
Smartphone use (hours/day)	4.26 ± 3.17

BMI: body mass index.

## Data Availability

Not applicable.
